# Novel Approaches in Molecular Imaging and Neuroimaging of Fibromyalgia

**DOI:** 10.3390/ijms232415519

**Published:** 2022-12-08

**Authors:** Maria Ricci, Andrea Cimini, Maria Rosaria Grivet Fojaja, Mariacristina Ullo, Bruno Carabellese, Viviana Frantellizzi, Ennio Lubrano

**Affiliations:** 1 Nuclear Medicine Unit, Cardarelli Hospital, 86100 Campobasso, Italy; 2 Nuclear Medicine Unit, St. Salvatore Hospital, 67100 L’Aquila, Italy; 3 Department of Radiological Sciences, Oncology and Anatomo-Pathology, Sapienza University of Rome, 00161 Rome, Italy; 4 Department of Rheumatology, University of Molise, 86100 Campobasso, Italy

**Keywords:** positron emission tomography, fibromyalgia, functional MRI, molecular imaging

## Abstract

Fibromyalgia (FM) represents a condition that is still controversial in its entity, pathophysiology, diagnosis and management. The aim of this review is to focus on imaging aspects of FM, especially on novel approaches in molecular imaging, with a special focus on neuroimaging. Novel functional and molecular imaging findings may represent, eventually, future biomarkers both in research settings and in terms of clinical practice. Several imaging techniques have already been tested in clinical trials in the FM field, including functional MRI, positron emission tomography (PET) imaging with ^18^F-FDG in FM, PET imaging of the dopaminergic system, PET imaging of the GABAergic system, PET imaging with neuroinflammation and neuroimmune parameters, PET imaging of the opioid system and H_2_^15^O-PET activation studies. Therefore, the potential role in the FM field of fMRI and different PET tracers has been discussed in different settings, serving as a comprehensive guide of novel imaging options both in research and in the clinical field.

## 1. Introduction

Fibromyalgia is defined by chronic widespread pain with further symptoms, such as joint stiffness, fatigue, and symptoms related to possible central nervous system (CNS) alterations, such as sleep disturbance, cognitive dysfunction, and depression. Fibromyalgia (FM) is also defined by widespread pain in the absence of identifiable peripheral pathology and, according to the American College of Rheumatology (ACR) criteria, FM diagnosis is based exclusively on a comprehensive clinical assessment [1]. However, validated and confirmed biological and imaging biomarkers associated with FM have not yet been identified. In fact, according to the modified 2016 FM criteria [2], the diagnosis of FM is confirmed if the following conditions are met:

(1) Widespread pain index (WPI) of Z 7 and symptom severity scale (SSS) score of Z 5 or WPI of 4–6 and SSS score of Z 9.
(2) Generalized pain (the pain must be described in at least four out of five regions, excluding jaw, chest, and abdominal pain).
(3) The symptoms described should be present for at least 3 months.
(4) A diagnosis of fibromyalgia is valid irrespective of other diagnoses.

However, the diagnosis of FM does not exclude the presence of other diseases [2].

Whereas widespread musculoskeletal pain, tenderness, fatigue and further peripheral symptoms may be the hallmark symptoms of FM, the eventual presence and entity of cognitive dysfunction caused by further symptoms associated with CNS alterations may have a significant impact on patient management [3]. FM represents a condition that is still controversial and its entity, including pathophysiology, diagnosis and management, still represents a matter of debate among physicians. In fact, beyond the clinical criteria, there is no gold standard for the diagnosis of FM, and this aspect represents a challenge, especially considering the heterogeneity of symptoms and the clinical overlap with other disorders. Therefore, the FM field is characterized by misconnection and a lack of specific biomarkers; therefore, FM is considered as a subjective experience, as patients often receive normal test and analysis results, without any objective biomarkers that can be identified by physicians or relatives [4]. Genetic and environmental factors have been considered in FM pathogenesis and FM perpetuation has been explained by an alteration in the nociceptive system, thus leading to a neuroendocrine syndrome of chronic stress [5]. In fact, genome-wide association studies have investigated the genes potentially involved in FM pathogenesis, highlighting that genetic factors are possibly responsible for disease susceptibility; a gene–environmental interaction has been proposed as a triggering mechanism through epigenetic alterations [1]. A previous study proposed a selection of five strikingly downregulated miRNAs to be used as biomarkers of FM, but validation in multi-centric trials with larger samples is needed in order to consider these findings in the clinical practice [6]. In addition, previous papers have considered the role of subclinical inflammation and endothelial dysfunction in the pathophysiology of fibromyalgia. Based on these aspects, the authors selected potential markers for FM, such as serum endocan levels [7], and potential biomarkers of therapy monitoring [8]. In addition, in FM patients, significantly low serum serotonin levels [9] or altered metabolic profiles, evaluated by urine analysis, have been reported [10]. Moreover, a further paper suggested the possible correlation of high leptin levels, independent of adiposity, with the clinical entity of FM symptoms in women with FM [11]. A previous paper reports that gut microbiome analysis combined with serum metabolomics can provide novel insights into the FM pathogenesis background [12]. Furthermore, a proteomic analysis reported that the interplay of the complement and coagulation cascades contributes to the inflammatory process, by identifying two proteins, haptoglobin and fibrinogen, as potential biomarker candidates of FM for future studies [13]. Further potential biomarkers have been evaluated by using vibrational spectroscopy [14] or by using laser-evoked potentials (LEPs) and paired laser stimuli, describing objective findings obtained from increasingly conditioned Aδ-LEP amplitudes and supporting the potential role of hyperexcitability in the pain matrices in FM patients [15].

Despite all these potential biomarkers, validated biological and imaging biomarkers associated with fibromyalgia have not yet been identified. Therefore, although previous research has provided a significant amount of data, FM still represents an emblematic condition of unexplained and subjective medical symptoms and some physicians remain doubtful about its reality and entity [16]. Nevertheless, FM generally has a very significant impact on the daily life of the patient in several aspects (social, work, family, etc.), especially considering that therapeutics (aerobic exercise, antidepressants and antiepileptic drugs) show minimal effectiveness [5]. Researchers aim to achieve a diagnosis, in order to be able to manage the FM in a realistic way for the patient. Although the precise pathophysiology of FM is still a subject of debate, most FM subjects display diffuse hyperalgesia/allodynia, which is identifiable through both quantitative sensory testing and functional imaging [17].

Therefore, the aim of this review is to focus on imaging aspects of FM. We will explore the role of molecular and functional imaging, particularly PET imaging and functional imaging in FM patients, in order to provide an overview of the potential imaging biomarkers of FM, with a special focus on neuroimaging. In fact, even through various peripheral and spinal mechanisms have been hypothesized to contribute to pain amplification and chronicity, the potential role of the CNS in the FM field remains to be fully elucidated. Functional brain imaging techniques, such as positron emission tomography (PET) and functional magnetic resonance imaging (fMRI), have frequently been used to investigate brain activity regarding pain perception, in particular acute pain or pain during experimental tasks, helping to expand the knowledge of the human pain network. Therefore, central nervous system alterations associated with pain have been extensively studied with neuroimaging techniques and, despite the variability in patients and methodologies, there is a growing consistency of data regarding the involvement of several brain regions in pain processing. On the other hand, this review will assess the potential role of novel molecular imaging approaches through peripheral evaluation. The final purpose of this review is to identify the molecular and functional diagnostic techniques that have already been tested in the literature for FM patients in clinical trials, with a special focus on the possible FM molecular changes that lead to the imaging findings, serving as a comprehensive guide of novel imaging techniques for physicians in clinical and research settings. All the extracted studies were subgrouped based on the imaging technique used and, then, all the results obtained in each imaging category were analyzed and discussed with a special focus on the possible physio-pathological changes that underlie the imaging results, with the final aim of forming a basis for the growing knowledge on imaging techniques for FM patients. An overview of the main findings included in the review is provided in Table 1.

## 2. Novel Imaging Approaches to Fibromyalgia

### 2.1. fMRI in Fibromyalgia

Magnetic resonance imaging (MRI) may be useful in order to assess morphological changes in the central nervous system (CNS) of patients with FM, regarding white and grey matter [53]; Jensen et al. [54] reported a significant decrease in white matter in some specific areas of FM patients, such as the left rostral anterior cingulate cortex and the left lateral orbitofrontal cortex. Regarding the grey matter in these patients, an increase in some specific regions (such as the orbitofrontal cortex, cerebellum, basal ganglia, cingulate cortex and insula) and a decrease in other areas (such as the superior temporal gyrus, thalamus, amygdala, and periaqueductal grey) have been found in several studies [55,56,57].

In the FM field, neuroimaging MRI techniques have led to the study of abnormal processing through different aspects, such as quantitative sensory testing, neural networks, neurotransmitter evaluation, and morphological alterations in brain regions [58]. Tonic activation related to the presence of ongoing widespread pain can be supported and implemented by both a facilitation increase and inhibition decrease in pain modulation networks [58]. A previous fMRI study identified that patients with FM had increased periaqueductal grey connectivity with the insula, anterior cingulate cortex, and anterior prefrontal cortex. Periaqueductal grey functional connectivity correlated with pain severity, disease duration, and the depressive personality trait rating, supporting the hypothesis of an endogenous pain modulatory system dysfunction in FM [18].

Functional magnetic resonance imaging (fMRI) seems to be an interesting tool in the management of fibromyalgia, but the results are heterogeneous. Notably, a previous paper analyzed the subjective experience of cognitive dysfunction (“fibrofog”), objective cognitive task performance, and brain activity during a cognitive task by using fMRI, describing no significant neurological correlates [19]. However, a further paper analyzed the differences between control datasets and FM datasets, describing that the model-driven analyses did not identify differences in pain processing between the HC and FM patients, whereas the data-driven analyses identified significant group differences in both datasets [20], suggesting that further and standardized analysis models are needed in order to define the potential neuro biomarkers in the FM field.

On the other hand, changes in brain activity in FM patients using fMRI have been demonstrated, including increased activity in the insula, cerebellum, amygdala and thalamus [59,60]. The cerebellum may play a key role in the pain assessment of these patients, since the activation of this region is correlated with catastrophizing scores [61]. Conversely, decreased activity has been found in the brainstem, thus suggesting an altered central pain regulatory system in patients with FM [54].

Moreover, an interesting previous fMRI study that aimed to correlate network activity to translocator protein (TSPO) regulation revealed that a TSPO high-affinity binding group had more pronounced pain-evoked functional connectivity in the right frontoparietal network, between the dorsolateral prefrontal area and the parietal cortex [21]. 

Interestingly, resting-state fMRI may provide further data concerning the possible alteration in the power spectral density (PSD) of low-frequency fluctuations in brain regions associated with central pain modulation. A previous paper that focused on this topic in FM patients described atypical increased frequency power during the resting state in pain-related brain regions [22].

Similarly, some authors have focused on the role of fMRI in the peripheral assessment of the brainstem and spinal cord [23], describing significant differences in brainstem/spinal cord network connectivity between the FM and healthy controls, which correlated with subjective differences in pain responses. Other authors used fMRI to study brainstem and spinal cord activation during temporal summation of second pain (TSSP), characterizing the time course of spinal cord and brainstem BOLD activity during TSSP; an alteration in brainstem activity was reported in patients with FM, possibly caused by deficient pain modulation [24].

Magnetic resonance spectroscopy (MRS) may be also useful in order to assess brain metabolites in these patients, with modifications in the levels of glutamate, choline and N-acetylaspartate found in the hippocampus, insula and prefrontal cortex [62]. With regard to glutamate, Fayed et al. also found an increase in this metabolite in the posterior cingulate cortex of fibromyalgia patients, suggesting a potential role of glutamate in the molecular processes of the disease. In addition, the same research group has shown by means of proton magnetic resonance spectroscopy (H-MRS) that the reduction in glutamatergic levels with pharmacological treatment may improve the symptomatology of these patients [25].

### 2.2. PET Imaging in Fibromyalgia

#### 2.2.1. ^18^F-FDG Imaging in Fibromyalgia

The role of CNS alterations in FM is, therefore, largely discussed [58] and PET with ^18^F-FDG may provide novel data concerning glucose metabolism both in neuroimaging and in peripheral assessment. Previous findings have demonstrated that pain-related non-dermatomal somatosensory deficits, which present with replicable neuroperceptive clinical findings, showed a complex pattern in ^18^F-FDG PET neuroimaging. However, the authors described a hypometabolic pattern in the changes in the cortical and subcortical areas that were not directly correlated to pain indexes (a visual analogue scale pain score and Hamilton Depression score) but, according to the authors, were related to the condition [63].

Moreover, a reduction in grey matter density has been described in FM patients. Therefore, the reduction in grey matter density can be considered as a neuroimaging biomarker, even if the exact mechanisms that lead to these findings are not clear yet. It has been proposed that thalamic dysfunction, defined both as a morphology alteration (reduction in grey matter) and as a functional alteration (hypoperfusion), may lead to different types of chronic pain syndromes [55,58], including FM. A previous paper concerning the metabolism of 18 treatment-naïve FM subjects suggests an association between the metabolism in the thalamus, lentiform nucleus, and parahippocampal gyrus and prognosis [26]. Nevertheless, insular hypometabolism has also been described in a case report of a patient with FM [27]. A further paper described no significant differences in ^18^F-FDG uptake between patients and controls for all the brain structures measured [28], but these heterogeneous results may be explained by the different methods and equipment. A further approach, concerning peripheral assessment, includes the evaluation of brown adipose tissue (BAT) by using ^18^F-FDG PET/CT. BAT activity was the same under warm conditions in both healthy controls and FM patients, attesting to the reproducibility and reliability of BAT quantification by using ^18^F-FDG PET [29]. In addition, concerning BAT assessment, ^18^F-FDG PET imaging has also been performed to assess the BAT adaptation to cold stimulus in order to evaluate eventual differences in their sympathetic responses. When measured at standard temperature (24 °C), four parameters, such as blood pressure (BP), skin temperature, serum glucose, and BAT activity, analyzed via ^18^F-FDG PET imaging did not differ between the controls and individuals with FM. However, after a reduction in temperature exposure (19 °C), BP and BAT activity increased in the controls but not in the FM subjects; skin temperature on the calf and arm decreased in healthy subjects more than in FM subjects, and circulating glucose was lower in FM subjects than in healthy subjects. Pain sensitivity did not change during the testing interval in response to cold. These findings strongly suggest that individuals with FM have impaired sympathetic responses to stress that are observable and highly significant [30].

Furthermore, beyond the potential role of ^18^F-FDG PET imaging in the assessment of neurological and peripheral changes in FM, a potential interesting role is played by therapy monitoring, both in research and in clinical settings.

A previous ^18^F-FDG PET study aimed to monitor the modulation of brain metabolism in several limbic structures after multidisciplinary rehabilitation, describing an increase in limbic metabolism with concomitant symptomatic improvement. The possible implications of these findings include the hypothesis that the limbic system attenuates FM symptoms [31]. A double-blind, randomized, placebo-controlled study investigated the impact of repetitive transcranial magnetic stimulation (rTMS) on the quality of life (QoL) of patients with FM, which is associated with the post-treatment improvement of QoL with a concomitant increase in right limbic glucose metabolism, demonstrating the need for a neural substrate to offset the impact of rTMS on emotional dimensions involved in QoL [32].

Similarly, PET investigations with ^18^F-FDG have been performed in order to evaluate the role of supplementation with CoQ10, with promising results [33].

#### 2.2.2. PET Imaging of the Dopaminergic System

The etiology of FM is largely unknown. However, several factors appear to underlie the disorder, including the dysfunction of the central nervous system, neurotransmitters and autonomic nervous systems [34,64]. Furthermore, growing evidence suggests that the mesolimbic dopamine (DA) system modulates the perception of nociceptive processes and the symptoms associated with chronic pain [65]. Therefore, accumulating evidence shows that different types of chronic pain, including the pain experienced by FM patients, leads to alterations in dopamine metabolism [66]. In addition, dopamine is involved in the descending inhibitory modulation of pain transmission, which is an additional link between hyperdopaminergia and chronic pain [34,67].

Therefore, there is a growing interest in dopamine metabolism assessment, with different PET tracers, which may reveal changes in dopaminergic states. In particular, there are some interesting findings regarding the 6-[(18)F]fluoro-L-DOPA (^18^F-DOPA) biodistribution in the FM field. A semiquantitative PET analysis revealed reductions in ^18^F-DOPA uptake in several brain regions, indicating a disruption of presynaptic dopamine activity, wherein dopamine plays a potential key role in natural analgesia [35]. In an extension of this study, a positive correlation was demonstrated between an index of dopamine metabolism from the ventral tegmental area, wherein cell bodies of corticolimbic projection neurons originate, and grey matter density, specifically in the bilateral parahippocampal gyri and left pregenual cortex [36]. Finally, a recent paper described the effectiveness of Mindfulness-Oriented Recovery Enhancement (MORE) intervention in restoring dopamine function, reducing pain, and improving mood symptoms, by evaluating ^18^F-DOPA biodistribution [34].

Several papers have focused on the characterization of the dopamine system in individuals with FM by using further neuroimaging tools, including novel PET tracers. Dopamine D2/D3 receptor availability has been evaluated by using a novel radiotracer, ^11^C- raclopride [37]. In fact, a recent comparative study between FM patients with and without comorbid depression evaluated the ratio of specifically bound non-displaceable radio-compounds at equilibrium (BP(ND)) as a measure of D2/D3 receptor availability, describing differences among the compared groups in striatal regions (left ventral striatum, left caudate nucleus and left nucleus accumbens) and also showing differences between the groups concerning clinical indices [37]. 

In addition, an interesting aspect of PET imaging is that it can also be performed in a task-related modality. Particularly, several papers have evaluated the DA metabolism during tasks, which are as follows.

A task-related study was performed in order to determine the endogenous release of dopamine, by using ^11^C-raclopride (D2/D3 ligand) PET imaging in response to a pain task and by comparing the uptake during the injection of painful hypertonic saline and non-painful normal saline. The fibromyalgia patients reported that the hypertonic saline was more painful than the healthy controls. In contrast to FM patients, the control subjects released dopamine in the basal ganglia during the painful stimulation, and the dopamine release correlated with the perceived pain, a finding that was not present in the FM group data [38].

Moreover, the ^11^C-raclopride PET bolus-plus-infusion method was used to measure the ^11^C-raclopride receptor binding potential between an unpredictable reward condition and a sensorimotor control condition, with the aim to investigate the role of serotonin (5-HT) in the control of central dopamine systems, and their dysfunction in FM patients, suggesting a potential role of serotonin polymorphism in DA receptor modulation [39]. Furthermore, ^18^F-fallypride (FAL) is a further example of a suitable PET compound for the investigation of extrapyramidal D(2)-receptors, also bonding to D(2)-receptor sites, as shown by the preclinical findings [68]. PET with FAL has been used in order to assess DA response during a working memory task relative to a baseline task and, in addition, in order to evaluate eventual associations between baseline D2/D3 availability and pain indices. In patients with FM, self-reported spontaneous pain negatively correlated with FAL distribution at baseline in the left orbitofrontal cortex and parahippocampal gyrus. The baseline imaging results were significantly negatively correlated with experimental pain sensitivity and tolerance in both FM patients and control subjects, even if the spatial patterns of these associations differed among the groups. Therefore, these findings suggest that changes in DA metabolism may be associated with differential processing of pain perception in FM [40].

#### 2.2.3. PET Imaging of the GABAergic System

PET using the tracer ^18^F-flumazenil yields quantitative voxel-wise, whole-brain information on cortical GABAA receptors. Flumazenil binds to the benzodiazepine site of GABAA (but not GABAB) receptors, which are expressed at inhibitory synapses in the cortex [41,69], but few studies are available for FM clinical settings.

In a paper focused on ^18^F- flumazenil PET, the authors found decreased grey matter in FM to be associated with T1 relaxation times, a surrogate marker of water content, but not with GABA receptor concentration, a surrogate marker of neuronal integrity. In contrast, regional grey matter increases can be partially explained by the GABA receptor concentration, suggesting a possible form of neuronal plasticity [41]. A further flumazenil investigation reported increased pain sensitivity, impaired immediate memory, and increased cortical GABAA receptor concentration in the attention and default-mode networks, which were positively correlated with the functional scores and current pain in areas that overlapped with regions of increased GABAA receptor concentration in FM post-menopausal women [42].

#### 2.2.4. PET Imaging of Neuroinflammation and Neuroimmunity

Neuroinflammation, a further potential key actor in the FM field, can be explored with PET imaging by using, as a biomarker, the distribution volume ratio (DVR) of ^11^C-(R)-(2-chlorophenyl)-N-methyl-N-(1-methylpropyl)-3-isoquinoline-carboxamide (^11^C-(R)-PK11195). ^11^C -(R)-PK11195 is a novel PET tracer used for its property to bond to a translocator protein (TSPO), which is expressed by activated microglia or astrocytes [70]. A previous report described the abnormal ^11^C-(R)-PK11195 uptake, considered as a marker of neuroinflammation levels, in the brains of FM patients, with respect to patients with complex regional pain syndrome [43]. PET/CT investigations have also been performed by using a further tracer, ^11^C-PBR28, which also binds to a translocator protein (TSPO) that is upregulated in activated microglia and astrocytes. In a previous paper on ^11^C-PBR28, in an attempt to disentangle the contributions of different glial cell types to FM, a smaller sample was scanned at KI with ^11^C-deprenyl-D2 PET that was thought to primarily reflect astrocytic (but not microglial) signals. The authors reported in their exploratory, uncorrected analyses that higher subjective ratings of fatigue in fibromyalgia patients were associated with higher ^11^C-PBR28 SUVRs in the anterior and posterior middle cingulate cortices, suggesting a glial modulation in FM physiopathology [44].

A further approach includes the evaluation of cerebral blood flow with ^15^O-butanol PET as a measure of immune function and natural killer cell activity. However, this approach has been tested only in a small group of patients (5 FM) and, to our knowledge, this method has not been tested in further papers. However, according to the authors, natural killer cell activity was negatively correlated with the right hemisphere activity in the secondary somatosensory and motor cortices, as well as in the thalamus. Furthermore, natural killer cell activity was negatively and bilaterally related to activity in the posterior cingulate cortex, suggesting a relation between the immune parameters and the activity in brain areas involved in pain perception, emotion, and attention [45].

#### 2.2.5. PET Imaging of the Opioid System

Further developments may regard the role of mu, delta, and kappa opioid receptors in pain, as well as potential applications of PET imaging in the evaluation of FM and further chronic pain conditions [45]. Particular emphasis has been placed in the evaluation of endogenous opioid system tone on regional pain-evoked brain activity in a population characterized by chronic pain [17]. Previous studies have shown that increased m-opioid receptor (MOR) binding potential (BP) is associated with reduced pain sensitivity and more effective endogenous analgesia in healthy controls [71,72]. Moreover, a further paper described the heterogeneity in endogenous opioid system functional indicators across different pain conditions, and alterations in both receptor availability and endogenous opioid system activity in patients with chronic non-specific back pain [46], supporting the crucial role of the opioid system in chronic pain conditions. The binding of endogenous opioids to MOR can be indirectly detected with the selective radiotracer ^11^C-carfentanil; therefore, PET imaging allows the evaluation of a combination of MOR density and MOR affinity [17]. 

The initial data concerning FM revealed the decreased central mu-opioid receptor availability within several regions known to play a role in pain modulation, including the nucleus accumbens, the amygdala, and the dorsal cingulate [47].

In addition, previous evidence supports the hypothesis that dysfunction of the endogenous opioid system in FM could lead to less excitation in antinociceptive brain regions via incoming pain stimulation, which may lead to the hyperalgesia and allodynia frequently described in FM [17]. PET with ^11^C-carfentanil has also been used in the therapy monitoring of FM patients subjected to traditional Chinese acupuncture, suggesting a potential role of this methodology in this setting [48].

Moreover, a further PET tracer has been tested for this purpose, the opioid receptor ligand F-18- fluoro-ethyl-diprenorphine (^18^F-FEDPN), providing further evidence that a reduction in cortical opioid receptor availability in FM patients represents another potential central nervous system contributor to pain in FM [49].

#### 2.2.6. PET H_2_ ^15^O Activation Studies

The combination of PET with H_2_ ^15^O studies may provide further data concerning both the brain and peripheral evaluation for chronic pain conditions, such as FM. In fact, a recent comparative paper on patients with irritable bowel syndrome with and without FM evaluated the regional cerebral blood flow using this methodology. The authors suggested that chronic stimulus-specific enhancement of anterior cingulate cortex responses to sensory stimuli may play a key pathophysiologic role in these chronic pain syndromes [50].

Recently, occipital nerve field stimulation has been proposed as a potential treatment for FM-related pain, and cerebral blood flow assessment with PET imaging has been performed in order to monitor the therapy during both the “ON” (active stimulation) and “OFF” (stimulating device turned off) conditions. PET shows that occipital nerve field stimulation may act via activation of the descending pain inhibitory pathway and the lateral pain pathway in FM, while EEG shows activation of those cortical areas that could be responsible for descending inhibition system recruitment [51]. 

Furthermore, a previous H_2_ ^15^O- PET study was performed in order to evaluate the impact of transcranial magnetic simulation with promising results and also described an interesting and reproducible protocol for therapy monitoring in FM patients [52].

## 3. Discussion

The novel imaging tools used in FM management may provide further information concerning the physiopathological aspects of FM, the novel promising research fields and, evidently, the clinical management of the disease. In fact, according to the scientific literature and occasionally in clinical practice, fibromyalgia causes a controversial debate between physicians, which is described in some cases as an inexplicable clinical condition without objective signs [16]. Therefore, the evaluation of novel imaging biomarkers and indicators may provide a significant benefit to FM patients, both in terms of research and in terms of clinical settings. However, the main aspect of FM is that for a similar stimulus, FM patients experience a greater subjective sensation of pain. It has been suggested that prolonged nociceptive input to the brain might induce functional and morphologic changes and modulate processes that, in turn, further exacerbate the experience of chronic pain [73]. These aspects are considered to be central in the comprehension of the physiopathology of FM and neuroimaging tools may also contribute to the comprehension of the physiopathology of neurological conditions. FM is hypothesized as a prolonged stress consequence, affecting the modulation of brain functions via the hypothalamic–pituitary–adrenal axis and the sympathetic nervous system. Augmented subjective pain may be related to an increase in the corticotropin-releasing hormone, substance P, and glutamate in cerebrospinal fluid and salivary cortisol [58]. Moreover, in FM patients, several alterations in neurotransmission activities have been described, including dopaminergic, opioidergic, and serotoninergic systems, which may lead to increased pain sensitivity and inhibition of serotonin synthesis [74]. The main aim of this review was to provide a comprehensive description of recent imaging approaches tested in FM patients in different settings that are able to support physicians in the elaboration and validation of research protocols.

Concerning the potential role of fMRI in FM, fMRI seems to be an interesting tool in the management of FM, but, of course, further multi-centric trials are needed in order to clearly define the potential role of this imaging tool in FM management. In fact, the results published so far are heterogeneous and not definitive. However, some reports have described the promising role of fMRI in the eventual assessment of neurological changes in FM, with a special focus on the eventual specific pattern of network connection in FM patients that may include changes in activity in the insula, cerebellum, amygdala and thalamus [59,60,61]. However, different authors have described changes in the fMRI findings in the brainstem [23,24,54]. In addition, further studies have reported that peripheral assessment may provide further findings concerning the potential alteration in spinal cord findings [23,24]. Further data are needed for the evaluation of magnetic resonance spectroscopy (MRS) in FM research and clinical management.

Concerning the potential role of ^18^F-FDG PET/CT in FM, neuroimaging studies have demonstrated differential involvement of a variety of brain regions in fibromyalgia, including the evaluation of the glucose metabolism in the brain regions. Therefore, ^18^F-FDG PET/CT may be an interesting tool in FM assessment. Most papers regarding the role of ^18^F-FDG are focused, in fact, on neuroimaging and on the metabolism in brain regions. Further multi-centric trials are needed in order to define a specific metabolic pattern for FM diagnosis, but some authors reported changes in brain metabolism in FM regions in small groups of patients [26,27,58,63]. However, the results are not heterogeneous [28] and the usage of ^18^F-FDG PET in clinical and research settings is not yet clearly defined. On the other hand, there is a growing interest in peripheral glucose metabolism assessment in the FM field, especially considering the potential role of ^18^F-FDG PET in the evaluation of BAT activity, which may be considered as an indicator of dysregulation in thermoregulation and in the processing of pain [29]. This hypothesis needs further confirmation. However, some authors have agreed upon the possible usage of ^18^F-FDG PET in therapy response assessment. The published reports regarding these aspects differ in the treatments used, purposes and methodology [30,31,32,33]. However, considering the growing interest in the patient-tailored and personalized approach and also considering the relative absence of confirmed imaging biomarkers in the FM field, it may be suitable to include ^18^F-FDG PET imaging in therapy monitoring in dedicated research settings.

The role of the dopamine system in fibromyalgia is still a subject of debate, and further developments indicate the role of dopamine in nociception and cognitive processing in patients with chronic pain. Different authors have reported that FM patients have an abnormal dopamine response to pain, both in rest assessment and in task-related assessment [34,37]. Therefore, the changes in dopaminergic reactivity in FM could be an important factor underlying the widespread pain and discomfort in FM patients. Moreover, different lines of evidence suggest that the therapeutic effects of dopaminergic treatments for this intractable disease should be explored [38]. The mentioned data provide support for the disruption of dopaminergic neurotransmission in FM and implicate DA as an important neurochemical moderator of differences in pain perception, while also considering clinical subgroups of FM patients with and without comorbid depression [37]. However, even if the role of DA in FM is interesting and the novel developments in the PET imaging field allow the quantification of DA tracer distribution, the role of DA in FM has not yet been defined. Therefore, the possible role of PET tracers in the dopamine system is still related to dedicated research settings, with a special focus on improving our knowledge of the neurological mechanisms that lead to the clinical symptoms. Therefore, the role of PET imaging in DA evaluation of FM is promising, especially considering the possible improvement of the physiopathology background. However, the mentioned papers include small samples and validation through multi-centric trials is needed.

Concerning the role of GABAergic system assessment in FM, few reports have described alterations in FM patients, possibly indicating an imbalance between excitatory and inhibitory neurotransmission [41,42]. Future studies should try to understand the nature of the dysregulation of the GABAergic system in FM and in other pain syndromes.

Abnormal neuroinflammation can be also an important pathological factor in FM. Furthermore, glial modulation seems to be a potential therapeutic strategy for FM [44]. The initial results suggested that abnormal neuroinflammation might be an important player in FM physiopathology. Therefore, the identification of common and different critical regions related to abnormal neuroinflammation in FM may contribute to improved diagnosis and, potentially, to the development of novel therapies for patients with FM [43]. Even if the current research is at the initial stage, neuroinflammation and neuromodulation may be a novel promising area in FM analyses and management. 

The interest in the opioid system in FM physiopathology is growing and PET imaging allows the evaluation of mu-opioid receptor availability using novel radiotracers. The data concerning these aspects are both scarce and heterogeneous; however, the potential role of ^18^F-FEDPN and ^11^C-carfentanil imaging in pain physiopathology evaluation, including conditions of chronic pain such as FM, is very promising in the research setting [17,49].

The H_2_^15^O-PET activation studies are very interesting, especially in terms of therapy monitoring [50,51,52], but there are not enough data to provide useful information. 

## 4. Conclusions

The findings described are heterogeneous and multicenter trials are needed in order to clarify the role of fMRI and PET imaging with different tracers in FM. However, in the context of a growing interest in a personalized and tailored approach to a patient, especially with a disease as controversial as FM, the possibility to use different imaging approaches for different purposes may open new chapters in the research setting, particularly those focused on the analysis of the disease’s background and on novel therapies’ development and assessment. 

Potentially, by using different methodologies, several aspects of FM may be explored, such as specific patterns of network connection in FM, eventual changes in glucose metabolism, the eventual role of the DA, GABAergic and opioid systems in FM, and the potential impact of neuroinflammation. The applications of novel imaging approaches for FM patients in research settings may provide useful information about the physiopathology of FM, leading eventually to the validation of novel biomarkers with potential future application also in clinical practice. 

## Figures and Tables

**Table 1:** Overview of reports included in the review.

Authors	Year	Imaging Technique	Numbers of Patients	Title	Main Results
Schrepf A. et al. [17]	2016	fMRI; ^11^C-carfentanil PET/CT	18 female FM patients	*Endogenous opioidergic dysregulation of pain in fibromyalgia: a PET and fMRI study*	The obtained data suggest that dysregulation of the endogenous opioid system in FM could lead to less excitation in antinociceptive brain regions by incoming noxious stimulation, resulting in the hyperalgesia and allodynia commonly observed in this population.
Truini A. et al. [18]	2016	fMRI	20 FM patients; 15 HC	*Abnormal resting state functional connectivity of the periaqueductal grey in patients with fibromyalgia*	Abnormal resting-state functional connectivity of the periaqueductal grey suggests that patients with fibromyalgia have endogenous pain modulatory system dysfunction, possibly causing impaired descending pain inhibition.
Walitt B. et al. [19]	2016	fMRI	16 FM patients; 13 HC	*Characterizing “fibrofog”: Subjective appraisal, objective performance, and task-related brain activity during a working memory task*	The subjective experience of cognitive dysfunction (“fibrofog”) appears to be better characterized by subjective, rather than objective, impairment. Neurologic correlates of this subjective experience of impairment might be separate from those involved in the performance of cognitive tasks.
Warren H.J.M. et al. [20]	2021	fMRI	First dataset (15 FM patients vs 15 HC); second dataset (15 FM patients vs 11 HC)	*How fMRI Analysis Using Structural Equation Modeling Techniques Can Improve Our Understanding of Pain Processing in Fibromyalgia*	Consistent with previous studies, the model-driven analyses did not identify differences in pain processing between the HC and FM patients, but the data-driven analyses identified significant group differences in both datasets.
Kosek E. et al. [21]	2016	fMRI	*24 fMRI imaging patients*	*The translocator protein gene is associated with symptom severity and cerebral pain processing in fibromyalgia*	Functional connectivity analyses revealed that the TSPO high-affinity binding group had more pronounced pain-evoked functional connectivity in the right frontoparietal network between the dorsolateral prefrontal area and the parietal cortex.
Kim J.Y. et al. [22]	2013	fMRI resting state	19 female FM patients; 20 female HC	*Increased power spectral density in resting-state pain-related brain networks in fibromyalgia*	Atypical increased frequency power during the resting state in pain-related brain regions may implicate the enhanced resting-state baseline neural activity in several brain regions associated with pain processing in FM.
Ioachim G. et al. [23]	2022	fMRI	15 FM patients; 15 HC	*Altered Pain in the Brainstem and Spinal Cord of Fibromyalgia Patients During the Anticipation and Experience of Experimental Pain*	The results demonstrate significant differences in brainstem/spinal cord network connectivity between the fibromyalgia and control groups, which also correlated with individual differences in pain responses. The regions involved in these differences in connectivity included the locus coeruleus, hypothalamus, periaqueductal gray matter, and parabrachial nucleus, which are known to be associated with autonomic homeostatic regulation, including fight or flight responses.
Staud R. et al. [24]	2021	fMRI	14 FM patients; 16 HC	*Spinal cord neural activity of patients with fibromyalgia and healthy controls during temporal summation of pain: an fMRI study*	HC and FM participants had similar temporal patterns of spinal activation, including an initial BOLD increase followed by deactivation. Structural equation modeling showed that the observed spinal activity during temporal summation of second pain (TSSP) was associated with higher BOLD activity across/within the brainstem in FM subjects than HC, suggesting differences in pain modulation.
Fayed N. et al. [25]	2012	MRI MRS	10 FM patients; 10 patients with somatization disorder; 10 HC	*Brain dysfunction in fibromyalgia and somatization disorder using proton magnetic resonance spectroscopy: a controlled study*	Glutamate seems to be relevant in the molecular processes involved in fibromyalgia and somatization disorder. It is also associated with proton MRS (^1^H-MRS) in somatization disorder and the results suggests that reducing glutamatergic activity through pharmacological treatment could improve the outcome of patients with fibromyalgia and somatization disorder.
Usui C. et al. [26]	2017	^18^F-FDG PET/CT	18 FM patients; 18 HC	*A study of brain metabolism in fibromyalgia by positron emission tomography*	The present findings suggest an association between the metabolism in the thalamus, lentiform nucleus, and parahippocampal gyrus and prognosis in FM patients.
Wood P.B. et al. [27]	2008	^18^F-FDG PET/CT	1 FM patient	*Insular hypometabolism in a patient with fibromyalgia: a case study*	The analysis of the scans revealed metabolic hypoactivity within the left insular cortex.
Yunus M.B. et al. [28]	2004	^18^F-FDG PET/CT	12 FM patients; 7 HC	*Positron emission tomography in patients with fibromyalgia syndrome and healthy controls*	^18^F-FDG-PET scan findings for FM patients were not significantly different from those for healthy controls.
Pardo J.V. et al. [29]	2017	^18^F-FDG PET/CT	13 FM patients; 11 HC	*Automated quantitation of cold-inducible human brown adipose tissue with FDG PET/CT with application to fibromyalgia*	BAT recruitment can be quantified non-invasively with ^18^F-FDG PET using semi-automated techniques in human subjects across different diagnostic groups or within groups undergoing manipulations of interest.
Walitt B. et al. [30]	2007	^18^F-FDG PET/CT	9 FM patients	*The effects of multidisciplinary therapy on positron emission tomography of the brain in fibromyalgia: a pilot study*	After multidisciplinary treatment, a clinical improvement was noted in FM patients. Following treatment, increases in brain metabolism were noted in various components of the limbic system. An increase in limbic metabolism was noted with concomitant symptomatic improvement, suggesting that the limbic system attenuates FM symptoms.
Pardo J.V. et al. [31]	2019	^18^F-FDG PET/CT	13 FM patients; 11 HC	*Exposure to Cold Unmasks Potential Biomarkers of Fibromyalgia Syndrome Reflecting Insufficient Sympathetic Responses to Stress*	The convergence of the effect of cold on 4 relatively simple measures of thermogenic, cardiovascular, and metabolic activity, each regulated by sympathetic activity, strongly indicates that individuals with FM have impaired sympathetic responses to stress that are observable and highly significant even when measured in extraordinarily small sample populations. If insufficient sympathetic responses to stress are linked to FM, stress may unmask and maximize these potential clinical biomarkers of FM and be related to its etiology.
Boyer L. et al. [32]	2014	^18^F-FDG PET/CT	38 FM patients	*rTMS in fibromyalgia: A randomized trial evaluating QoL and its brain metabolic substrate*	The study shows that rTMS improves the QoL of patients with fibromyalgia. This improvement is associated with a concomitant increase in the right limbic metabolism, indicating the need for a neural substrate to offset the impact of rTMS on emotional dimensions involved in QoL.
Sawaddiruk P. et al. [33]	2019	^18^F-FDG PET/CT	11 FM patients	*Coenzyme Q10 supplementation alleviates pain in pregabalin-treated fibromyalgia patients* via *reducing brain activity and mitochondrial dysfunction*	These findings provide new evidence that CoQ10 supplementation offers further benefits for relieving pain sensations in pregabalin-treated FM patients, possibly via improving mitochondrial function.
Ledermann K. et al. [34]	2021	^18^F-DOPA PET/CT	64 FM patients	*Understanding and restoring dopaminergic function in fibromyalgia patients using a mindfulness-based psychological intervention: a [18F]-DOPA PET study. Study protocol for the FIBRODOPA study-a randomized controlled trial*	If the findings of this study confirm the effectiveness of Mindfulness-Oriented Recovery Enhancement (MORE) intervention in restoring dopamine function, reducing pain, and improving mood symptoms, MORE can be confirmed as a promising means to improve the quality of life of FM patients.
Wood P.B. et al. [35]	2007	^18^F-DOPA PET/CT	6 female FM patients; 8 HC	*Reduced presynaptic dopamine activity in fibromyalgia syndrome demonstrated with positron emission tomography: a pilot study*	FM might be characterized by a disruption in dopaminergic neurotransmission.
Wood P.B. et al. [36]	2009	^18^F-DOPA PET/CT	30 female FM patients; 20 HC	*Changes in gray matter density in fibromyalgia: correlation with dopamine metabolism*	FM is associated with altered brain morphometry. Alterations in dopamine metabolism might contribute to the associated changes in grey matter density.
Ledermann K. et al. [37]	2016	^11^C-raclopride PET/CT	11 FM patients with comorbid depression; 13 FM patients without comorbid depression; 17 HC	*Relation of dopamine receptor 2 binding to pain perception in female fibromyalgia patients with and without depression--A [¹¹C] raclopride PET-study*	These findings provide further support for the disruption in dopaminergic neurotransmission in FM and implicate DA as an important neurochemical moderator of differences in pain perception in FM patients with and without comorbid depression.
Wood P.B. et al. [38]	2007	^11^C-raclopride PET/CT		*Fibromyalgia patients show an abnormal dopamine response to pain*	These findings provide the first example of direct evidence that fibromyalgia patients have an abnormal dopamine response to pain.
Ledermann K. et al. [39]	2020	^11^C-raclopride PET/CT	23 female FM patients; 17 HC	5’*UTR polymorphism in the serotonergic receptor HTR3A gene is differently associated with striatal Dopamine D2/D3 receptor availability in the right putamen in Fibromyalgia patients and healthy controls-Preliminary evidence*	These preliminary results indicate that polymorphisms (SNPs) in the serotonin receptor-3 rs1062613 in the serotonergic receptor HTR3A gene possibly modulate the striatal dopamine D2/D3 receptor availability.
Albrecht D.S. et al. [40]	2016	^18^F-FAL PET/CT	12 female FM patients; 11 HC	*Differential dopamine function in fibromyalgia*	The data suggest that abnormal DA function may be associated with differential processing of pain perception in FM.
Pomares F.B et al. [41]	2017	^18^F-flumazenil PET/CT	26 female FM patients; 25 HC	*Histological Underpinnings of Grey Matter Changes in Fibromyalgia Investigated Using Multimodal Brain Imaging*	Regional grey matter decreases were largely explained by T1 relaxation times in grey matter, a surrogate measure of water content, and not to any substantial degree by the GABA receptor concentration, an indirect marker of neuronal integrity measured with (F) flumazenil PET.
Pomares F.B et al. [42]	2020	^18^F-flumazenil PET/CT	26 female FM patients; 25 HC	*Upregulation of cortical GABAA receptor concentration in fibromyalgia*	This study shows increased GABAA receptor concentrations in FM, which are associated with pain symptoms and impaired function. The changes were widespread and not restricted to pain-processing regions. These findings suggest that the GABAergic system is altered, possibly indicating an imbalance between excitatory and inhibitory neurotransmission.
Seo S. et al. [43]	2021	^11^C-(R)-PK11195 PET/CT	12 FM patients; 11 patients with complex regional pain syndrome (CRPS); 15 HC	*Abnormal neuroinflammation in fibromyalgia and CRPS using [11C]-(R)-PK11195 PET*	The results suggested that abnormal neuroinflammation can be an important pathological factor in FM. In addition, the identification of common and different critical regions related to abnormal neuroinflammation in FM, compared with patients with CRPS and healthy controls, may contribute to improved diagnosis and the development of effective medical treatment for patients with FM.
Albrecht D.S. et al. [44]	2018	^11^C-PBR28 PET/CT; ^11^C-L-deprenyl-D_2_ PET/CT	31 FM patients; 27 HC; 11 FM patients; 11 HC	*Brain glial activation in fibromyalgia*—*A multi-site positron emission tomography investigation*	The elevations in ^11^C-PBR28 signal were not accompanied by increased ^11^C-deprenyl-D2 signals and the data suggest that microglia, but not astrocytes, may drive the TSPO elevation in these regions. Although the ^11^C-L-deprenyl-D_2_ signal was not found to be increased in FM patients, larger studies are needed to further assess the role of possible astrocytic contributions in FM.
Lekander M. et al. [45]	2000	^15^O-butanol PET/CT	5 FM patients	*Neuroimmune relations in patients with fibromyalgia: a positron emission tomography study*	Immune parameters were related to activity in brain areas involved in pain perception, emotion, and attention. Taking into account the small study population, these strong neuro-immune associations are discussed in view of recent findings concerning mechanisms and adaptive values in immuno-cortical communication and regulation.
Martikainen I.K. et al. [46].	2013	^11^C-carfentanil PET/CT	16 patients with chronic non-specific back pain; 16 HC	*Alterations in endogenous opioid functional measures in chronic back pain*	During both pain expectation and sustained pain challenges, patients with chronic non-specific back pain showed regional reductions in the capacity to activate this neurotransmitter system compared with the control sample, which are further associated with clinical pain and affective state ratings.
Harris R.E. et al. [47]	2007	^11^C-carfentanil PET/CT	17 FM patients; 17 HC	*Decreased central mu-opioid receptor availability in fibromyalgia*	FM patients display reduced MOR binding potential (BP) within several regions known to play a role in pain modulation, including the nucleus accumbens, the amygdala, and the dorsal cingulate. MOR BP in the accumbens of FM patients was negatively correlated with affective pain ratings. Moreover, MOR BP throughout the cingulate and the striatum was also negatively correlated with the relative amount of affective pain within these patients.
Harris R.E. et al. [48]	2009	^11^C-carfentanil PET/CT		*Traditional Chinese acupuncture and placebo (sham) acupuncture are differentiated by their effects on mu-opioid receptors (MORs)*	Long-term increases in MOR binding availability following acupuncture were associated with greater reductions in clinical pain. These findings suggest that divergent MOR processes may mediate clinically relevant analgesic effects following acupuncture and sham acupuncture.
Üçeyler N. et al. [49]	2020	^18^F-FEDPN	7 FM patients	*Cortical Binding Potential of Opioid Receptors in Patients with Fibromyalgia Syndrome and Reduced Systemic Interleukin-4 Levels*—*A Pilot Study*	Our data give further evidence for the reduction in cortical opioid receptor availability in FM patients as another potential central nervous system contributor to pain in FM.
Chang L. et al. [50]	2003	H_2_^15^O PET/CT	10 irritable bowel patients (IBS); 10 IBS + FM patients	*Brain responses to visceral and somatic stimuli in patients with irritable bowel syndrome with and without fibromyalgia*	Group differences in regional brain activation were recorded within the middle subregion of the anterior cingulate cortex. There was a greater regional cerebral blood flow increase in response to noxious visceral stimuli in IBS patients and to somatic stimuli in IBS + FM patients.
Ahmed S. et al. [51]	2018	H_2_^15^O PET/CT	7 FM patients implanted with subcutaneous electrodes in the C2	*The effect of occipital nerve field stimulation on the descending pain pathway in patients with fibromyalgia: a water PET and EEG imaging study*	H_2_15O PET scans were taken during both the “ON” (active stimulation) and “OFF” (stimulating device was turned off) conditions. PET shows that occipital nerve field stimulation exerts its effect via activation of the descending pain inhibitory pathway and the lateral pain pathway in fibromyalgia, while EEG shows activation of those cortical areas that could be responsible for descending inhibition system recruitment.
Tzabazis A. et al. [52]	2013	H_2_^15^O PET/CT	16 FM patients; 16 HC	*Shaped magnetic field pulses by multi-coil repetitive transcranial magnetic stimulation (rTMS) differentially modulate anterior cingulate cortex responses and pain in volunteers and fibromyalgia patients*	Repetitive transcranial magnetic stimulation may be a safe and effective treatment option for acute as well as for chronic pain, such as that accompanying fibromyalgia. Further studies are necessary to optimize the configurations and settings, as well as to elucidate the mechanisms that lead to the long-lasting pain control achieved by these treatments.

FM: fibromyalgia; HC: health controls; fMRI: functional magnetic resonance imaging; MRS: magnetic resonance spectroscopy; TSPO: translocator protein; ^18^F-FDG: 18F-fluorodeoxyglucose; PET: positron emission tomography; PET/CT: positron emission tomography/computed tomography; BAT: brown adipose tissue; DA: dopamine; ^18^F-DOPA: ^18^F-fluoro-L-DOPA; ^18^F-FAL: (18)F-fallypride; ^11^C-(R)-PK11195: 11C-(R)-(2-chlorophenyl)-N-methyl-N-(1-methylpropyl)-3-isoquinoline-carboxamide; MOR: mu-opioid receptor; ^18^F-FEDPN: F-18- fluoro-ethyl-diprenorphine.
